# Population characteristics and diagnosis rate of chronic kidney disease by eGFR and proteinuria in Japanese clinical practice: an observational database study

**DOI:** 10.1038/s41598-024-55827-7

**Published:** 2024-03-02

**Authors:** Tetsuhiro Tanaka, Shoichi Maruyama, Noriharu Chishima, Hiroki Akiyama, Koji Shimamoto, Shoichiro Inokuchi, Keiji Yokota, Asuka Ozaki

**Affiliations:** 1 Department of Nephrology, Rheumatology and Endocrinology, Tohoku University Graduate School of Medicine, Sendai, 980-8574 Japan; 2 Department of Nephrology, Nagoya University Graduate School of Medicine, Nagoya, 466-8550 Japan; 3 Medical Affairs, AstraZeneca K.K., Osaka, 530-0011 Japan; 4 Research and Analytics Department, Real World Data Co., Ltd., Kyoto, 600-8233 Japan

**Keywords:** Nephrology, Kidney diseases, Epidemiology

## Abstract

Chronic kidney disease (CKD) guidelines recommend early identification and intervention to delay the progression of CKD. The Kidney Disease: Improving Global Outcomes (KDIGO) heatmap is widely used for risk evaluation in CKD management; however, real-world evidence on clinical characteristics based on the KDIGO heatmap remains limited worldwide including Japan. In order to understand the management of CKD including its diagnostic rates in a Japanese clinical setting on the basis of KDIGO heatmap, we utilized a medical record database that contains estimated glomerular filtration rate (eGFR) and urine protein data. Adult individuals (≥ 18 years) with two eGFR results of < 90 mL/min/1.73 m^2^, 90–360 days apart, were included. Approximately half of patients (452,996/788,059) had proteinuria test results and 6.9% (54,073) had quantitative results. CKD diagnosis rate in patients without proteinuria data was 5.9%, with a lower rate (2.9%) in stage G2; the corresponding rates with quantitative test results were 43.5% and 31.3%, respectively. The most frequent comorbidities were hypertension, diabetes, and cardiovascular disease, and their prevalence increased as the eGFR and proteinuria stages progressed. This study revealed a low rate of proteinuria assessment, especially using quantitative methods, and diagnosis in individuals with suspected CKD. With emerging treatment options to prevent CKD progression and complication onset, there is a need for early evaluation and diagnosis of CKD.

## Introduction

Chronic kidney disease (CKD) imposes a large burden on the life expectancy and the quality of life (QoL) of patients^1^. Worldwide, 700 million individuals (9.1% of the population) have CKD, and in Japan, 13 million individuals (13% of the adult population) have CKD, and this number is increasing^2,3^. The progression of CKD is associated with a broad range of adverse clinical consequences such as cardiovascular diseases, hospitalizations, and mortality^4–6^. Some patients with CKD eventually progress to end-stage renal disease (ESRD) where dialysis or kidney transplantation (renal replacement therapy) is required. This results in a reduction in patient QoL and an increased medical resource consumption. In Japan, the annual survival rate of patients on dialysis has been reported to be alarmingly low (40% at 60 months) in 2010, and the cost of dialysis was 1.62 trillion JPY in 2018^7,8^.

The risk of developing renal and cardiovascular events, ESRD, and mortality is substantially high even in patients in early stages of the disease with an estimated glomerular filtration rate (eGFR) of 60 mL/min/1.73 m^2^ and no apparent albuminuria (urinary albumin-to-creatinine ratio [UACR] < 30 mg/g)^9^. Furthermore, an earlier referral to a nephrologist has been reported to result in better clinical outcomes^10^. To delay the progression of CKD and reduce the incidence of clinical events and complications^11,12^, early identification and intervention are recommended in the Japanese Society of Nephrology (JSN) guidelines as well as in the internationally recognized Kidney Disease: Improving Global Outcomes (KDIGO) guidelines.

Although many studies report risk prediction models that incorporate several demographic parameters, medical histories, and/or laboratory measures for patients with CKD^13,14^, the KDIGO risk classification (hereafter referred to as “KDIGO heatmap”) by the two factors eGFR (mL/min/1.73 m^2^) and albuminuria (mg/gCr) is widely used for risk evaluation to determine treatment strategy for patients with CKD^8^. Several previous studies reported characteristics and prognosis of patients with CKD for each eGFR stage^15,16^. However, little evidence is available in Japan on the prevalence, characteristics, treatment patterns, and clinical outcomes of patients with CKD based on the KDIGO heatmap, mainly because of the challenges of finding both eGFR and quantitative urinary protein data in databases available in Japan.

The Real World Data database (RWD-DB) used in the present study has electronic medical record data of both inpatients and outpatients and contains data on laboratory tests, diagnoses, and prescriptions. This DB allowed us to categorize patients using a KDIGO heatmap based on eGFR and quantitative/semi-quantitative proteinuria test results, albeit for a limited number of patients, ~ 20% of Japanese population. This study assessed the prevalence and characteristics of patients with CKD, and the diagnostic rate of CKD in each KDIGO heatmap category. Our study will contribute to understanding the current extent of under diagnosis and uncover unmet needs as an initial step in understanding ways to improve the diagnosis, management, and outcomes of CKD.

## Methods

### Study design and DB

This study retrospectively analyzed an anonymized medical record DB in Japan, the RWD-DB, maintained by the Health, Clinic, and Education Information Evaluation Institute (HCEI; Kyoto, Japan) and Real World Data Co., Ltd. (Kyoto, Japan). The DB comprises inpatient and outpatient EMR collected from approximately 200 medical institutions across Japan since 2000. The dataset includes information on the demographics, hospital diagnoses, prescriptions, medical procedures, and laboratory test results of approximately 23 million patients as of 2021.

Patients with two eGFR results less than 90 mL/min/1.73 m^2^ at least 90 days apart within 360 days (eGFR definitive period) were selected and the date of second qualifying eGFR measurement was defined as the index date. A cut of eGFR < 90 mL/min/1.73 m^2^ was set as the inclusion criteria following clinical trials that assessed efficacy and safety of medications for CKD and DKD^17–21^. The index date was between January 1, 2004, and September 30, 2021. Patients aged ≥ 18 years with medical records from 360 days prior to the index date (look-back period) were included.

Cross-sectional analyses were performed for 2005, 2010, 2015, and 2020, with each period starting on January 1 and ending on December 31 (the analytical period). Patients who had at least one eGFR datum in each period were extracted, and those with both eGFR and urine albumin/protein data on the same date were categorized according to the KDIGO heatmap, and the others were only categorized into the eGFR stages. If more than one value was obtained during the analytical period, the earliest value was used.

As this study used de-identified data, the requirement for informed consent was waived, and an opt-out approach was applied in compliance with local regulations. This study followed the principles of the Declaration of Helsinki. This study and waiver of informed consent were approved by the Ethics Committee of the Research Institute of Healthcare Data Science (Tokyo, Japan; approval number: RI2021018).

### Definition of CKD and measurements

Diagnosis of CKD was defined as the presence of at least one of the International Classification of Diseases 10th revision (ICD-10) codes for CKD (Supplementary Table S1) between 360 days before and 90 days after the index date. Patients who did not fulfill the CKD criteria, that is, patients categorized as G2 without proteinuria test results or G2A1, were excluded from the analyses of diagnostic rates, except for the analysis performed in patients without proteinuria data. Comorbidities were defined by the presence of ICD-10 codes at or before the index date. Medication use was defined as the presence of prescriptions during the lookback period (Supplementary Table S1). Medical procedures (Supplementary Table S1), all-cause hospitalizations (extracted from admission records in the Electric Medical Records [EMR] or Format 1 File in the Diagnosis Procedure Combination [DPC])^22^, and the latest laboratory measurements (extracted from the EMR) during the look-back period were used for analyses.

Patients with serum potassium (sK) levels < 1.0 or > 8.0 mmol/L were excluded from the analysis owing to potential laboratory errors. The highest value was used in the analysis if multiple results were obtained on the same date. Hyperkalemia was defined as sK > 5.0 mmol/L^23^. For other laboratory tests, the median value was used if multiple results were available. If multiple test results were available during the look-back period, the data closest to the index date were used.

### Categorizations into KDIGO heatmap

Patients were categorized into the KDIGO heatmap using following procedures.

Patients were divided into the following five eGFR stages based on their eGFR levels that were maintained for ≥ 90 days on the index date: G3a (< 60), G3b (< 45), G4 (< 30), G5 (< 15 mL/min/1.73 m^2^), and others as G2.

eGFR was calculated using a formula developed and recommended by the JSN^24^. If there were multiple values on the same day, the lowest serum creatinine level was used.

Patients with QPD were categorized based on eGFR and quantitative urinary albumin or protein data [daily urinary albumin (mg/day), UACR (mg/g creatinine), daily urinary protein (g/day), or urinary protein-to-creatinine ratio (UPCR, g/g creatinine]). The criteria used to define A1, A2, and A3 are listed in Supplementary Table S2.

Patients with APD were categorized using eGFR values and quantitative or semi-quantitative [urinary albumin/protein (dipstick) results (“(−),” “(+/−)” and ≥ “(+)”)] tests for urinary protein. Patients were divided into A1, A2, and A3 groups based on the level of urinary protein (Supplementary Table S2)^11^. If there were multiple results on the same day, the highest value or level of proteinuria was used.

### Statistical analysis

Categorical variables are presented as numbers and percentages. Continuous data are summarized as the number of non-missing observations and presented as mean ± standard deviation (SD) (in some cases, the median is in parentheses). All the analyses were performed using Python 3.7.9 (Python Software Foundation. Delaware, USA).

## Results

### Patient distribution, age and gender

**Figure 1:**
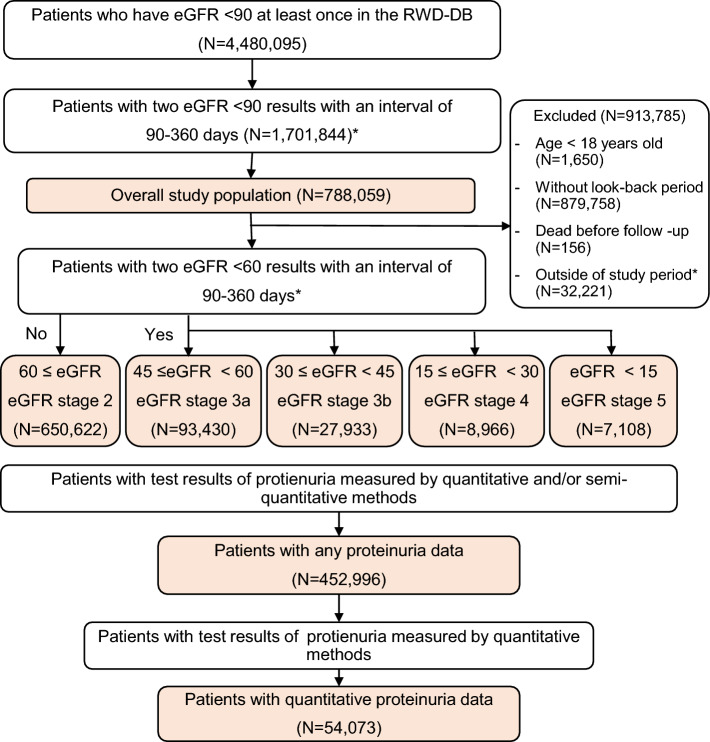
Patient flow chart for the study population and assignment of eGFR stages. *eGFR* estimated glomerular filtration rate (unit = mL/min/1.73 m^2^). *From January 1, 2004 to September 30, 2020.

A total of 788,059 patients with JSN eGFRcr (eGFR calculated using a formula developed and recommended by the JSN, see “Methods” section; referred to as eGFR) < 90 mL/min/1.73 m^2^ were included in the study (overall population). Of the overall population, test results of proteinuria measured using either semi-quantitative or quantitative methods were available for 452,996 patients (57.5% of overall population) (patients with any proteinuria data [APD group]). We further defined patients with quantitative proteinuria data (QPD; QPD group) when quantitative test results were available (54,073 patients, 6.9% of overall population) (Fig. 1). Distribution according to eGFR stage was comparable between the overall population and the APD group; however, QPD group had a higher proportion of patients with advanced CKD stages, such as G4 and G5 (Table 1).

**Table 1:**
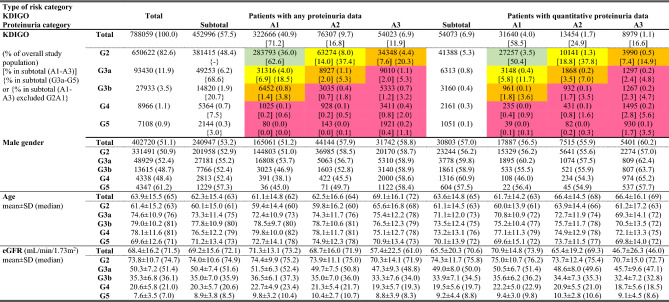
Subject demographics by the eGFR stage and the KDIGO heatmap.

Green; low risk, yellow; moderately increased risk, orange; high risk, red; very high risk. *eGFR* estimated glomerular filtration rate*, KDIGO* Kidney Disease: Improving Global Outcomes*, G* eGFR stage, *A* proteinuria categories.

A larger proportion of QPD patients were categorized into higher KDIGO risk categories compared with the proportion of APD patients. The patient distributions of APD/QPD in KDIGO heatmap were as follows: low (green cell in Table 1), 62.6/50.4%; moderate (yellow), 20.9/24.6%; high (orange), 11.0/12.6, and very high (red), 5.5/12.4%. For all CKD stages, more than half of patients were male, and the proportion of males tended to increase as the proteinuria category progressed from A1 to A3 (51.2%/57.9%/58.8%, and 56.5%/55.9%,/60.2% in APD and QPD, respectively). The mean age tended to increase from G2 to G4, with a gap of approximately 10 years between G2 and G3a and 2–3 years between G3a and G4. In contrast, the mean age of G5 was lower than that of G4.

### CKD diagnosis rate

Among patients fulfilling the CKD criteria of eGFR and/or proteinuria in the overall study population (235,059 patients), 16.9% (39,788 patients) had CKD diagnosis codes. Diagnosis rate varied depending on the availability and method of proteinuria testing; 16.5% of APD, 43.5% of QPD, and 5.9% of patients without proteinuria data were diagnosed with CKD.

**Figure 2:**
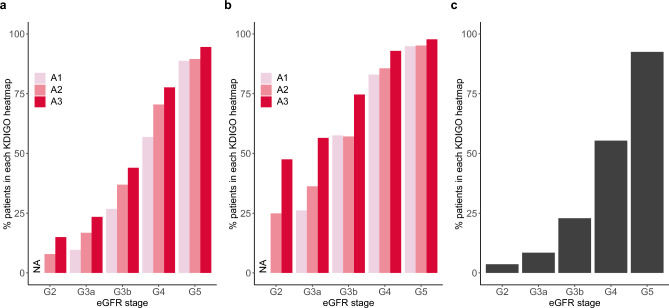
Diagnosis rate for chronic kidney disease in each KDIGO heatmap. (**a**) Patients with any proteinuria data, (**b**) Patients with quantitative proteinuria data, (**c**) Patients without proteinuria data. *eGFR* estimated glomerular filtration rate, *G* eGFR, *A* proteinuria category.

Of the overall study population, 650,622 patients were stage G2, and 381,415 patients (58.6%) had urinary protein test results. Among stage G2 patients, the CKD diagnosis rate in the population without proteinuria measurement data was 2.9% (Supplementary Table S3, Fig. 2), and corresponding values for the APD and QPD groups were 10.4% and 31.3%, respectively. When focusing on patients in moderate risk category (yellow cells in Supplementary Table S3), the diagnostic rate was 8.5% for the APD and 25.2% for the QPD.

Diagnostic rates were consistently high in the population with available proteinuria test data compared to those without, across all eGFR categories, with especially higher rates in the QPD group than in the APD group. Within the same eGFR category, the highest rate was found in category A3, and this trend was more evident in the early stages (G2 and G3a; Fig. 2). Most patients with advanced eGFR stages as stage G4 and G5 in both the APD and QPD groups were diagnosed with CKD. In contrast, only half of the stage G4 patients were diagnosed without proteinuria. The diagnosis rate decreased steeply during the early stages of the APD group, whereas a gentler decrease was observed in the QPD group.

Younger patients tended to be diagnosed at a higher rate than the older patients, and the diagnosis rate was particularly high in patients aged 18–49 years (Supplementary Fig. S1). The diagnosis rate for patients with complications (diabetes mellitus [DM], hypertension [HT], and heart failure [HF]) was higher than that for those without complications (Supplementary Table S3), except that a comparable rate was observed between patients with and without DM in the QPD group.

### Comorbidities

**Figure 3:**
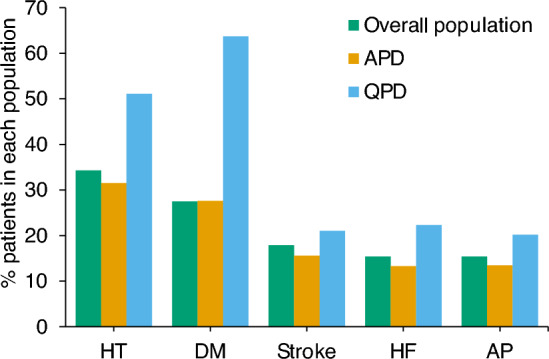
Percentage of comorbidities based on proteinuria data. *APD* patients with any proteinuria data, *QPD* patients with quantitative proteinuria data, *HT* hypertension, *DM* diabetes mellitus, *HF* heart failure, *AP* angina pectoris.

**Figure 4:**
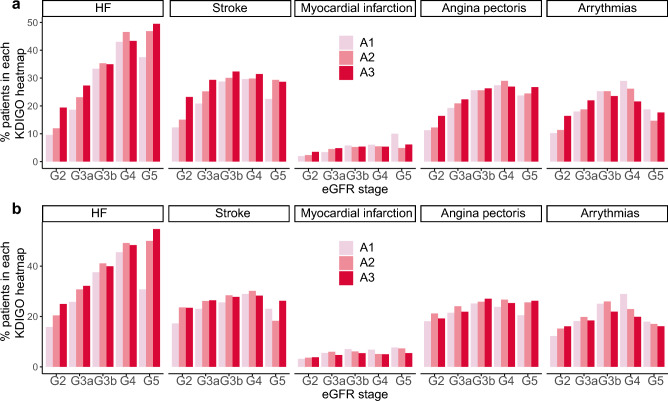
Prevalence of cardiovascular comorbidities in each KDIGO heatmap. (**a**) Patients with any proteinuria data. (**b**) Patients with quantitative proteinuria data. *HF* heart failure, *G* eGFR stage, *A* proteinuria category, *eGFR* estimated glomerular filtration rate.

The most frequently observed comorbidities were HT, DM, stroke, HF, and angina pectoris (Fig. 3, Supplementary Table S4). There was no apparent difference in the profile of major comorbidity prevalence between the APD and QPD groups, except for DM. The prevalence of HT tended to increase as the CKD stage progressed from G3 to G5 and proteinuria increased from A1 to A3 (Supplementary Table S4). The prevalence of DM was generally lower than that of HT in the APD group but higher in the QPD group, with a prevalence of more than 60% in A3 at all CKD stages. While an increased prevalence of DM with proteinuria category progression was observed in the APD group, no such trend was observed in G2 of the QPD group. In cardiovascular diseases (CVDs), the prevalence of stroke, myocardial infarction, angina pectoris, and arrhythmias increased as the CKD stage progressed from G3a to G4, whereas there was a lower association of urinary protein levels with the prevalence (Fig. 4). In contrast, the prevalence of HF increased as CKD progressed to G5 stage.

Hyperkalemia, defined by the sK value, increased as CKD and proteinuria progressed (Supplementary Table S4). The prevalence of hyperuricemia increased as the CKD and proteinuria increased.

### Medications

**Figure 5:**
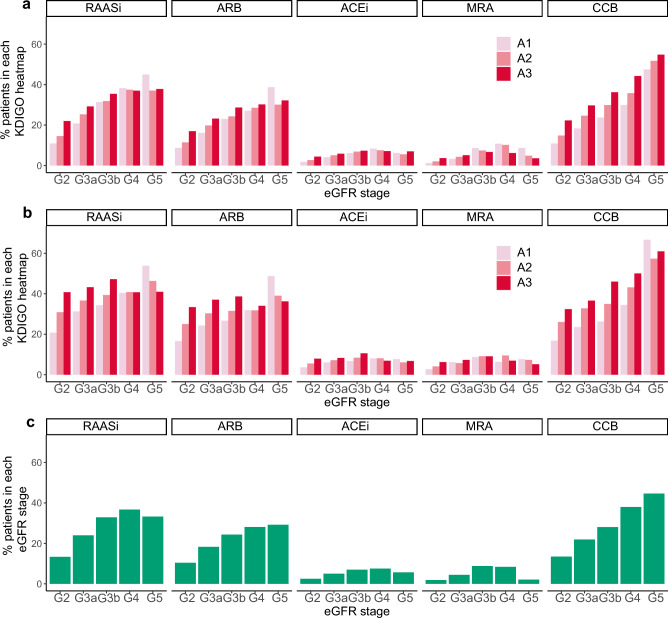
Prescription for Renin–angiotensin–aldosterone system inhibitors (RAASi) and Calcium channel blockers (CCB) in each KDIGO heatmap. (**a**) Patients with any proteinuria data. (**b**) Patients with quantitative proteinuria data. (**c**) Overall population. *ARB* Angiotensin II receptor blockers, *ACEi* angiotensin converting enzyme inhibitor, *MRA* mineralocorticoid receptor antagonists, *CCB* calcium channel blocker, *G* eGFR stage, *A* proteinuria category, *eGFR* estimated glomerular filtration rate.

Proton pump inhibitors (16.4%), renin–angiotensin–aldosterone system inhibitors (RAASi; 15.7%), calcium channel blockers (CCB; 15.5%), and antilipidemics (12.1%) were the most frequently prescribed medications in the overall study population (Supplementary Table S5, Fig. 5). This trend was also observed for both the APD and QPD groups, with a higher prescription rate in the QPD group. Angiotensin receptor blockers (ARBs) were the most commonly prescribed RAASi in all stages; this trend increased as the eGFR and proteinuria progressed (Fig. 5, Supplementary Table S5). The prescription rate of CCB also increased as the CKD stages and proteinuria levels progressed. The use of diuretics increased from G2 to G5, whereas the progression of proteinuria did not appear to have a significant effect on eGFR stage progression. The most frequently prescribed oral glucose lowering drugs in the overall study population were dipeptidyl peptidase 4 inhibitors (3.6%), followed by sulfonylureas (2.7%), and biguanides (2.5%). A similar trend was observed for the APD and QPD groups.

### Laboratory test results

The laboratory testing rates during the lookback period are listed in Supplementary Table S6. Almost all measurements were performed most frequently in the QPD group. The profiles of testing rates for the eGFR stages were similar among the overall study population and the APD and QPD groups, with the exception of urine protein testing. Semiquantitative tests were the most commonly used method in the overall study population (63.2%) to evaluate urine protein (Supplementary Table S6) and were performed in 99.3% and 94.4% cases in the APD and QPD groups, respectively. UACR was measured in 47.6% (25,716) patients in the QPD and in 6.5% (29,451) patients in the APD groups (Supplementary Table S6). UACR in the QPD group tended to decrease as the CKD stage progressed. In contrast, UPCR test results increased as the CKD stage progressed. Tests for assessing anemia (Iron, TIBC, Ferritin, EPO) were performed more frequently during the progressive CKD and proteinuria stages. The percentage of immunoglobulin measurements tended to increase as proteinuria levels progressed in all CKD stages, except for G5, where the measurements were performed at similar rates in patients in stages A2 and A3. Similar trends were observed for CRP and ANCA test results.

### The longitudinal profiles of patients with CKD

The annual population in the DB increased from 43,508 in 2005 to 279,306 in 2020 (Supplementary Table S7). With regards longitudinal trends of CKD diagnosis rate, while the distribution of patients in each KDIGO heatmap was similar each year, diagnosis rate of CKD increased slightly from 2005 to 2020 (8.9% to 11.1%). Mean age and prevalence of DM and HT in 2020 did not change significantly from that in 2010 (DM/HT: 35.0%/43.1%) to 2020 (DM/HT: 39.3%/43.7%).

## Discussion

This study is the first to describe the detailed distribution and clinical characteristics of patients based on the KDIGO heatmap in Japan, utilizing laboratory results for eGFR and quantitative and/or semiquantitative urine protein. Measuring urine protein is an essential step in evaluating patients with suspected CKD; however, in this study, > 90% of patients with eGFR below 90 mL/min/1.73 m^2^ did not undergo quantitative tests and > 40% did not undergo either quantitative or semi-quantitative tests. These findings suggest that (1) there was insufficient initial testing for urinary protein in patients with suspected CKD, and (2) abnormal results are not further investigated using quantitative testing. Diagnosis rate in patients without proteinuria testing was quite low (5.9%), while it was substantially high (43.5%) in the QPD group, demonstrating the crucial role of assessing urine protein by quantitative methods for appropriate diagnosis of CKD. Although a much higher number of patients were diagnosed with CKD when they had quantitative test results, more than half were left undiagnosed despite the fact that diagnosis is a mandatory part of medical management of CKD as a part of the insurance system in Japan.

The most frequently observed comorbidity in the overall population and the APD group in the present study was HT, followed by DM and CVD. This trend is consistent with previous reports from Japan and other countries^25–27^. Conversely, in the QPD group, the prevalence of DM exceeded that of HT. This may be because quantitative UACR measurements are reimbursed only for patients with DM in Japan and the renal function of patients with DM is closely monitored from the early stages. Overall, the prevalence of CVDs increased from G2 to G3b/G4. HF seemed to have the strongest association with CKD progression, and its prevalence increased linearly as eGFR progressed. This finding is consistent with the cardiorenal interaction in which the kidney and heart physiologically and pathologically affect each other^28–31^. We also found that common risk factors for cardiovascular and renal dysfunction became more prevalent as CKD progressed; 60% of patients in A3 were male, median age in G3b and G4 was 81 years, and the prevalence of HT and DM was higher in patients with advanced eGFR and proteinuria stages. These results further support the association between CKD progression and the development of CVD as well as the common issues that these two organ dysfunctions share, emphasizing the importance of early intervention to prevent both CVD onset and renal function deterioration.

CKD guidelines recommend early diagnosis and treatment to prevent the progression of CKD^11,12^; however, several studies reported that approximately 20–25% of the patients in stages G3a or worse have diagnosis code for CKD^32–34^. A lower rate, less than 10%, was observed in stage G3 in a previous study using Japanese DB^35^. Similar results were obtained in the present study with diagnosis rate of the overall study population of 16.9%. Substantially higher proportion of patients with proteinuria data were diagnosed with CKD compared to those without; the diagnostic rates in the APD and QPD groups were 16.5% and 43.5%, respectively, whereas only 5.9% of patients without proteinuria data were diagnosed with CKD. This suggests that evaluating urine protein, especially by quantitative methods, could potentially lead to a higher diagnosis rate and improved disease management^36^. Dipstick testing is readily available at local clinics, at large hospitals, and also annual physical checkups; however, quantitative tests are not as prevalent, and the reimbursement of UACR is limited to those with DM in Japan. When we focused on the difference in diagnostic rate between patients with and without DM, higher rate was observed in patients with DM in without proteinuria data population (26.9% vs. 13.5%), whereas the rate was equivalent in the QPD group (43.5% vs. 43.5%). This finding suggests that quantitative assessments result in considerable improvement in the CKD diagnosis rate in patients without DM, equal to or greater than in those with DM. Therefore, it is worth noting that the limitations in the reimbursement of UACR in Japan might be reconsidered.

Consistent with the previous results^35^, diagnosis rate in the early stages was alarmingly low in the present study; only 10% of patients in G2 and G3a of overall population were diagnosed. Despite that CKD can only be diagnosed by testing for proteinuria at this stage of patients, approximately 42% of G2 patients did not have urine protein data. Since the difference in diagnosis rate between the APD and QPD groups was large in G2 (10.4% vs. 31.3%), implementation of quantitative proteinuria testing may considerably improve diagnosis rate of patients during this early stage. Furthermore, considering that CVD such as HF and stroke begin to become more prevalent in early stages of G2 to G3b (HF, 12.6% to 36.6%; stroke, 15.8% to 31.4%), low rate of diagnosis in early stages may indicate that the window of opportunity to prevent complications is missed.

Besides the early stages, the older population is often overlooked in the management of CKD^37^. It is known that the kidneys atrophy with age and the number of glomeruli decreases^38^. Therefore, it is generally considered that deterioration of kidney function in older patients is due to “normal” aging; annual decline of eGFR could be − 2 mL/min/1.73 m^2^ per year in patients without CKD^39^. In this study, the diagnosis rate in the overall population aged 18–49 years remains unsatisfactory at 20.2%. Nonetheless, further low rates were observed in older population, indicating a clear need to increase awareness of diagnosing CKD and implementing therapy for this population.

Serum immunoglobulins (IgA, IgG, and IgM), CRP, and ANCAs were tested more frequently in G3b or worse stages than in the early stages of G2 and G3a, and the frequency was higher in patients tested for urinary protein, especially when using the quantitative method. The same trend was observed in tests that assessed proximal tubule dysfunction, anemia, and electrolyte imbalance. Furthermore, compared with the overall population, laboratory tests were performed more frequently in all eGFR stages of the QPD group, even if the patients were categorized as A1. These results suggest that a comprehensive evaluation of CKD is more likely to be performed in patients with severe CKD and/or with quantitative urinary protein testing.

The most commonly prescribed drugs RAASi and CCB are most likely manage blood pressure. ARB was the most prescribed RAASi, likely due to losartan having an indication for diabetic nephropathy in type 2 DM with HT and proteinuria. Despite evidence of delayed progression of CKD with proteinuria and improvement in CV outcomes in patients with CKD with RAASi, prescription rate of RAASi was low (24.0% of G3a in the overall study population and 49.2% in patients with HT) compared to that in the European countries (59.9% in patients with CKD with eGFR < 60)^40^, and it was similar to that of CCB (21.9% of G3a in the overall population; Supplementary Table S5). This relatively low rate of RAASi use may be due to the increased risk of hyperkalemia due to decreased urinary potassium excretion in these populations (prevalence of hyperkalemia in overall study population: 22.4%/29.6% in G4/G5; Table S4)^23^. JSN guideline 2018 recommended the use of ACEi, ARB, and CCB, as well as diuretics, for management of HT in patients with CKD, with a suggestion of switching ACEi or ARB to CCB if patients in G4/G5 exhibit hyperkalemia and/or eGFR decline with the use of ACEi or ARB. In addition, CCB are recommended as a first-line antihypertensive therapy in patients with CKD aged > 75 years. Another factor to be considered is the relatively low body mass index of the older Japanese patients who are less tolerant to antihypertensives. The use of diuretics increased as eGFR progressed, but no relationship was observed with the degree of proteinuria. This suggests the clinical use of diuretics for fluid control but not for the management of proteinuria.

Although we cannot emphasize too much the importance of treating the underlying conditions, new pharmacological options for directly treating CKD to slow its progression and improve outcomes are rapidly expanding. One such an example is sodium-glucose cotransporter-2 inhibitors (SGLT-2i); in Japan, the SGLT-2i dapagliflozin was approved in 2021 for the treatment of CKD with or without type 2 diabetes. SGLT-2i have been shown to improve renal and cardiovascular outcomes in patients with and without type 2 DM^41–17^. Dapagliflozin reduced the kidney-specific composite outcome across the KDIGO heatmap without heterogeneity^44^. The renoprotective effect of SGLT-2i has also been proven in patients with CKD of broad etiologies^18^. Furthermore, SGLT-2i reduced the risk of hyperkalemia and gout^45^, and its acceptable safety profile has been reported in older and other high-risk subpopulations for adverse events^46,47^. Recent research on SGLT-2i has also shown cost-effectiveness for cardiovascular and kidney benefits^48,49^, and it is expected to be a mainstay of management of all range of CKD. Recent large randomized controlled trials have shown the cardiovascular and renal benefits of mineralocorticoid receptor antagonists (MRA) in patients with CKD and type 2 DM^19^, and MRA was recently approved for the treatment of CKD with type 2 DM. Cardiorenal syndrome is encountered in daily clinical practice, and these results indicate the barriers to and opportunities for CKD management. With emerging therapeutic options, it is critical to achieve early evaluation and ensuing appropriate diagnosis of CKD, which would allow timely medical intervention to improve patient and societal outcomes.

This study has a few limitations due to the characteristics of the DB: the RWD-DB mainly consists of hospital data and only 10 clinics are included, which may not allow our results to fully reflect clinical practice in Japan^50^. The data are based on DPC claims and EMR, and the validity of the data depends on the original data recorded. Data from different facilities are not linked, and one patient can be counted multiple times if a record exists in different facilities. And also, it was not possible to rule out that the proteinuria detected was transient and not pathological. Finally, causal relationships could not be evaluated, which is a challenge faced by observational studies.

## Conclusion

A high proportion of patients who were at risk of CKD or met the definition of CKD were not tested for urinary protein and were not diagnosed with CKD, especially in the early stages of CKD, in this real-world study. Given the recent emergence of therapeutic options that target CKD, there is a clear need to evaluate and diagnose CKD at early stages to improve outcomes, thus improving patient QOL and reducing societal and financial burdens.

### Supplementary Information

Supplementary Figure S1.

Supplementary Tables.

## Data Availability

The data cannot be shared publicly because of the privacy policy of Real World Data Co., Ltd.
