# Supplementary material for: Population characteristics and diagnosis rate of chronic kidney disease by eGFR and proteinuria in Japanese clinical practice: an observational database study
Source: Sci Rep. 2024 Mar 2;14:5172. doi: 10.1038/s41598-024-55827-7 (PMC10908847; doi:10.1038/s41598-024-55827-7)
Supplement: Supplementary file 1 — Supplementary Figure S1. [file 41598_2024_55827_MOESM1_ESM.pdf]

Supplementary figures

Title:
Population characteristics and diagnosis rate of chronic kidney disease by eGFR and proteinuria in Japanese clinical practice: an observational database study

Authors:
Tatsuhiro Tanaka[1], Shoichi Maruyama[2], Noriharu Chishima[3], Hiroki Akiyama[3], Koji Shimamoto[4], Shoichiro Inokuchi[4], Keiji Yokota[4], Asuka Ozaki[3*]

Affiliations:
[1] Department of Nephrology, Rheumatology and Endocrinology, Tohoku University Graduate School of Medicine, Sendai, Japan
[2] Department of Nephrology, Nagoya University Graduate School of Medicine, Nagoya, Japan
[3] Medical Affairs, AstraZeneca K.K., Osaka, Japan
[4] Research and Analytics Department, Real World Data Co., Ltd., Kyoto, Japan

Corresponding author:
Asuka Ozaki

Address: AstraZeneca K.K, Grand Front Osaka, Tower B
3-1, Ofuka-cho, Kita-ku, Osaka City, 530-0011
E-mail: asuka.ozaki@astrazeneca.com

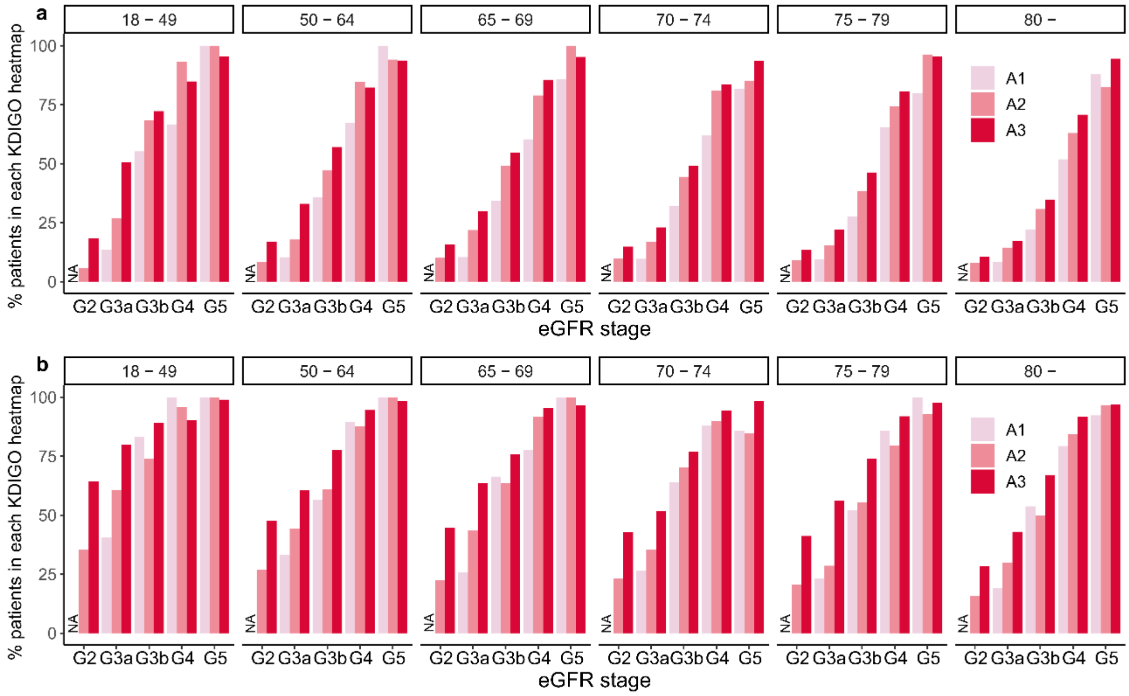

Supplementary Figure S1: Diagnosis rate for chronic kidney disease in each KDIGO heatmap by age group. (a) Patients with any proteinuria data. (b) Patients with quantitative proteinuria data. Abbreviations: *G* eGFR stage, *A* proteinuria category, *eGFR* estimated glomerular filtration rate, *NA* not applicable.
